# The biochar-based nanocomposites influence the quantity, quality and antioxidant activity of essential oil in dill seeds under salt stress

**DOI:** 10.1038/s41598-022-26578-0

**Published:** 2022-12-19

**Authors:** Kazem Ghassemi-Golezani, Saeedeh Rahimzadeh

**Affiliations:** Department of Plant Eco-Physiology, Faculty of Agriculture, University of Tabriz, Tabriz, Iran

**Keywords:** Environmental sciences, Plant sciences, Plant ecology, Plant physiology, Plant stress responses, Secondary metabolism

## Abstract

The essential oil content and composition of medicinal plants may be influenced by eco-friendly products for nutrient availability under abiotic stresses. This research was conducted to determine the effects of biochar (30 g kg^−1^ soil) and biochar-based nanocomposites (BNCs) of iron (30 g BNC-FeO kg^−1^ soil), zinc (30 g BNC-ZnO kg^−1^ soil), and their combined form (15 + 15 g) on dill (*Anethum graveolens* L.) under salinity levels (non-saline, 6 and 12 dS m^−1^). Application of biochar, particularly BNCs increased iron and zinc content and decreased sodium accumulation in leaf tissues. The seed essential oil content increased under high salinity. Salinity changed the values of major compounds in essential oil and induced the formation of compounds such as alpha,2-dimethylstyrene, cuminyl alcohol, *p*-cymene, and linalool. Biochar treatments especially BNCs with a higher production of monoterpenes increased the levels of limonene, carvone, apiol, and dillapioll. All extracts showed a considerable DPPH-inhibitory effect with application of BNCs under salinity. The maximum antioxidant activity was observed under high level of salinity with application of the combined form. Therefore, the combined form of nanocomposite was the best treatment to improve the content of basic commercial monoterpenes and consequently antioxidant activity of essential oil in salt-stressed dill plants.

## Introduction

Abiotic stresses create an imbalance in the environmental conditions that affect the normal growth and production of plants. Salinity as a major stress factor generally disrupts performance of medicinal plants by reducing the ability to maintain homeostasis^1^. Excessive uptake of ions such as sodium and chloride, nutritional imbalance, and the generation of reactive oxygen species leads to oxidative stress and notable limitation in plant growth^2^. Soil salinity can also affect the essential oil quantity and quality by changing its content and composition in medicinal plants^3^. Numerous secondary metabolites are produced by medicinal plants to assist a variety of cellular functions that are essential for plant defense response to physiological disturbances^4^. Saline conditions minimize the ability of plants to absorb water and nutrients that limit plant nutrition and reduce plant biomass^5,6^. Since enhancing yield and quality of essential oil have great commercial importance, some management strategies can be applied to reduce the negative impacts of salt stress and alter the secondary metabolites pathways in plants.

Application of biochar (an ideal way to recycle various agricultural wastes for sustainable development) enhances nutrient availability and soil quality that can reduce salt toxicity in plants^7–9^. Biochar is a solid carbon-rich material produced by pyrolysis (controlled process of thermal decomposition) under anoxic conditions and created useful characteristics, including high surface area and porosity, as well as high cation exchange capacity^10^. Biochar can alleviate salt toxicity in arid and semiarid soils and improve the growth and biochemical characteristics of plants^5,11,12^. Biochar has also an important role in immobilization of organic and inorganic contaminants^13^. Production of biochar-based nanocomposites can increase pristine biochar efficiency by combining the advantages of nanotechnology with biochar technology^6,14^. Biochar-based nanocomposites are the new classes of modified biochar materials with extraordinary improvement in porous structure, number of surface functional groups, and higher specific surface area^6,15^. Moreover, it is crucial to enhance nutrient availability for plants to improve their growth and productivity.

The biochar-based nutritional nanocomposites have been suggested as the efficient adsorbents of sodium ions in saline soils that help to improve soil fertility, nutrient availability, and growth of plants^6,16^. Macro and micro-nutrients as the components of all organic compounds are essential for normal physiological and biochemical processes of plants. The soil micronutrients such as zinc and iron have direct and major effects on biosynthesis and concentration of secondary metabolites, thus influencing the essential oil content and chemical composition of medicinal herbs^17–19^. In previous reports, using the nutrient-enriched biochar as a soil amendment and an alternative slow release fertilizer was beneficial for the plants nutrition^20–22^. Biochar-based nanocomposites with particular physicochemical properties and sodium adsorption capacity can positively affect nutrient absorption rate in poor-nutrient soils that finally lead to stress tolerance of plants^6,8^.

Dill (*Anethum graveolens* L.) is an annual aromatic herb belonging to the Apiaceae family. Essential oil production varies in different tissues of dill and is widely used by the food and pharmaceutical industries^23^. The chemical composition of dill essential oil (e.g. carvone, limonene, dillapiol) is widely used in the health industry as the antifungal, antinociceptive, anti-inflammatory, and is responsible for the aroma and biological effects^24,25^. Moreover, essential oil of dill plants with a great antioxidant activity plays an important role in neutralizing free radicals, thereby benefiting human health^26^.

Dill is more resistant to salinity than to water stress^27^. The highest percentage and yield of essential oil were obtained from dill seeds under stress conditions^23^. According to Ghassemi-Golezani et al.^28^ salinity caused an increase in percentage of constituents, and antioxidant activity of essential oil in dill seeds. In this way, the defense system and salt tolerance of plants improved^23^. Little is known about the response of dill seed essential oil to biochar-related treatments under saline conditions. Therefore, this research aimed to investigate the possible effects of biochar-based metal salts nanocomposites of iron and zinc on essential oil content of dill seeds, changes in secondary metabolites and antioxidant activity of dill seed essential oil in response to salt stress.

## Results

### Properties of biochar-based treatments

In response to the composition process, the physicochemical properties of biochar including the cation exchange capacity (CEC), pH, surface area (SBET), and porosity were enhanced. This process also increased sodium sorption capacity (Na-SC) of biochar by 72% and 131%, in iron and zinc nanocomposites, respectively. The contents of iron and zinc in biochar structure were increased in nanocomposite forms (Table 1). The particle sizes of nanocomposites including FeO and ZnO within the biochar matrix were 20–58 and 25–78 nm, respectively.

**Table 1:** Physicochemical properties of the experimental soil, biochar, and biochar-based nanocomposites of iron (BNC-FeO) and zinc (BNC-ZnO).

Soil		Biochar		BNC-FeO		BNC-ZnO	
pH	6.6	pH	7.7	pH	8.1	pH	7.9
CEC (cmol kg^−1^)	18.4	CEC (cmol kg^−1^)	19.5	CEC (cmol kg^−1^)	22.3	CEC (cmol kg^−1^)	24.2
Moisture (%)	< 1	Moisture (%)	< 1	Moisture (%)	< 1	Moisture (%)	< 1
N (%)	0.07	N (%)	0.41	N (%)	0.40	N (%)	0.35
P (mg kg^−1^)	45	P (mg kg^−1^)	5380	P (mg kg^−1^)	4910	P (mg kg^−1^)	5100
K (mg kg^−1^)	154	K (mg kg^−1^)	2360	K (mg kg^−1^)	2050	K (mg kg^−1^)	1800
Fe (mg kg^−1^)	8	Fe (mg kg^−1^)	230	Fe (mg kg^−1^)	1100	Fe (mg kg^−1^)	180
Zn (mg kg^−1^)	53	Zn (mg kg^−1^)	580	Zn (mg kg^−1^)	430	Zn (mg kg^−1^)	1490
Mg (mg kg^−1^)	43	Mg (mg kg^−1^)	210	Mg (mg kg^−1^)	202	Mg (mg kg^−1^)	180
EC (dSm^−1^)	1.6	Ca (mg kg^−1^)	4300	Ca (mg kg^−1^)	3760	Ca (mg kg^−1^)	4120
OC (g kg^−1^)	13	Mn (mg kg^−1^)	106	Mn (mg kg^−1^)	95	Mn (mg kg^−1^)	102
Texture	Silty loam	S (mg kg^−1^)	2670	S (mg kg^−1^)	2240	S (mg kg^−1^)	1630
		Na (mg kg^−1^)	4.7	Na (mg kg^−1^)	3.6	Na (mg kg^−1^)	4.0
		Cl (mg kg^−1^)	N. A	Cl (mg kg^−1^)	8.1	Cl (mg kg^−1^)	6.7
		C (%)	54	C (%)	52	C (%)	52
		H (%)	1.98	H (%)	2.23	H (%)	2.07
		O (%)	21.43	O (%)	23.10	O (%)	24.31
		S_BET_ (m^2^ g^−1^)	12.07	S_BET_ (m^2^ g^−1^)	114.75	S_BET_ (m^2^ g^−1^)	130.4
		V_tot_ (cm^3^ g^−1^)	0.031	V_tot_ (cm^3^ g^−1^)	0.173	V_tot_ (cm^3^ g^−1^)	0.042
		AVP (nm)	4.27	AVP (nm)	5.39	AVP (nm)	6.12
		Stability (°C)	540	Stability (°C)	515	Stability (°C)	525
		Na–SC (mg g^−^)	38	Na–SC (mg g^−1^)	65.4	Na–SC (mg g^−1^)	87.9

*EC* electrical conductivity, *OC* organic carbon, *CEC* cation exchange capacity, *S*_*BET*_ BET surface area, *V*_*tot*_ total pore volume, *AVP* average pore size, *Na–SC* sodium sorption capacity.

### Cations

**Figure 1:**
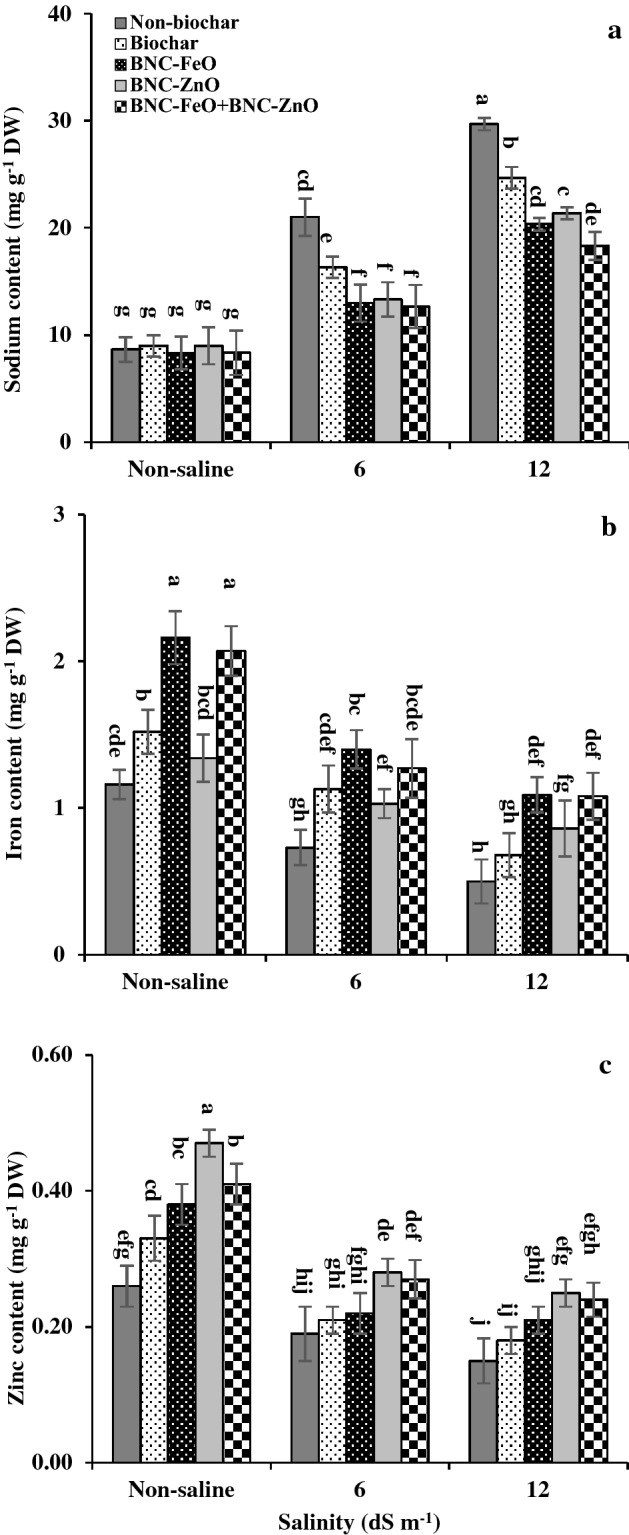
Means of sodium (**a**), iron (**b**), and zinc (**c**) contents in dill plants affected by biochar-based nanocomposites of iron and zinc under different levels of salinity. BNC-FeO: Biochar-based nanocomposite of iron; BNC-ZnO: biochar-based nanocomposite of zinc. The values are the means of three replicates ± SD (standard deviation). Different letters indicate significant differences by Duncan multiple range test at *p* ≤ 0.05.

Significant interaction of salinity and biochar-related treatments was observed for cations of dill plants (Fig. 1). Rising salt stress increased Na, but decreased Fe and Zn contents in dill leaf tissues. Biochar-related treatments had no significant effect on Na content under non-saline conditions, but these treatments reduced Na content under both moderate and high salinity levels. Biochar-based nanocomposites, had a better effect on reducing Na content of plants under saline conditions in comparison with pristine biochar. Under moderate stress, all of the nanocomposites similarly reduced Na content of plants. This reduction of Na by combined form of nanocomposites (BNC-FeO + BNC-ZnO) in comparison with pristine biochar was about 22% and 25% under moderate and high levels of salinity, respectively. Application of biochar forms led to an improvement in Fe, and Zn contents in both saline and non-saline conditions. Iron and zinc contents were increased in response to the biochar and BNCs. The highest iron content of plant tissues was recorded for BNC-FeO and combined treatments under non-saline and saline conditions. The BNC-FeO in comparison with the non-biochar treatment enriched the dill leaves with iron by about 91% and 118% under moderate and high level of salinities, respectively. Similar comparison of zinc content of dill plants due to BNC-ZnO and BNC-FeO + BNC-ZnO applications showed 47% and 42% increment under moderate stress, and 66% and 60% under high level of salinity, respectively.

### Seed essential oil

**Figure 2:**
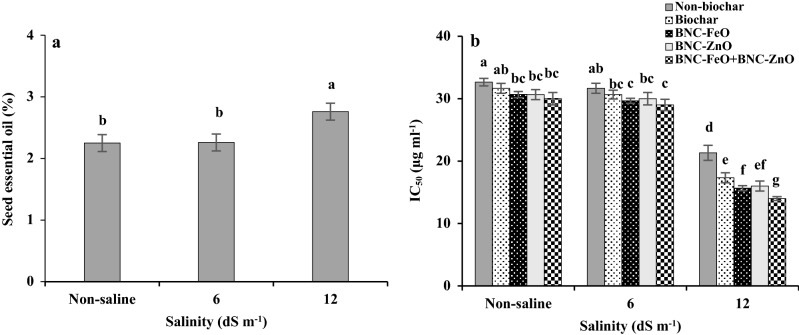
Changes in essential oil content of dill seed in response to salinity (**a**), and antioxidant activity (**b**) in dill seed affected by biochar-based nanocomposites of iron and zinc under different levels of salinity. BNC-FeO: Biochar-based nanocomposite of iron; BNC-ZnO: biochar-based nanocomposite of zinc; IC_50_: the essential oil concentration required to inhibit DPPH radical formation by 50%. The values are the means of three replicates ± SD (standard deviation). Different letters indicate significant differences by Duncan multiple range test at *p* ≤ 0.05.

The essential oil percentage of dill seeds was affected by salinity (Fig. 2a). The essential oil content of plants under non-saline conditions and moderate stress was statistically similar, while high level of salinity (12 dS m^−1^) caused an increase in essential oil content by about 23%.

### Chemical composition of essential oil

About 18 important constituents of seed essential oil were identified, representing 91.97–99.38% of the total essence content (Table 2). The GC/MS showed that carvone (45.16–53.77%), limonene (18.14–27.32%), dillapiol (12.12–16.78%) and apiole (2.32–3.11%) are the main constituents of dill seed essential oil. These chemicals varied among the salinity and biochar-related treatments. The limonene content decreased with increasing salinity level up to 12 dS m^−1^. The contents of carvone, apiol, dillapiole, and alpha-phellandrene in essential oil were enhanced with rising salt levels. Carvone was increased from 46.40% in non-saline condition to 51.40% in high level of salinity. The alpha,2-dimethylstyrene, cuminyl alcohol, *p*-cymene, and linalool were only detected in plants subjected to high salinity. In contrast, the production of sabinene in seed essential oil is restricted to non-saline condition.

**Table 2:** The chemical composition of seed essential oil of dill plants treated with biochar-based nanocomposites of iron (BNC-FeO) and zinc (BNC-ZnO) under different levels of salinity.

Compounds (%)	Non-saline					6 dS m^−1^					12 dS m^−1^				
	Non-biochar	Biochar	BNC-FeO	BNC-ZnO	BNC-FeO + BNC-ZnO	Non-biochar	Biochar	BNC-FeO	BNC-ZnO	BNC-FeO + BNC-ZnO	Non-biochar	Biochar	BNC-FeO	BNC-ZnO	BNC-FeO + BNC-ZnO
Beta-Pinene	1.11	1.20	1.52	1.37	1.34	1.05	1.20	1.26	1.32	1.38	1.06	1.20	1.14	1.22	1.22
Limonene	23.31	24.12	23.34	25.12	25.91	22.81	25.02	25.32	**27.32**	26.14	18.14	19.32	19.64	19.90	19.32
Carvone	46.40	46.27	46.77	45.16	45.87	47.00	47.32	48.05	47.38	47.10	51.40	52.47	52.59	52.85	**53.77**
Beta-Myrcene	0.32	0.35	0.34	0.36	0.34	0.33	0.35	0.33	0.37	0.45	0.24	0.25	0.28	0.24	0.27
Gamma-Terpinene	0.15	0.13	0.17	0.15	0.12	0.15	0.15	0.19	0.15	0.18	0.16	0.11	0.14	0.12	0.14
Apiol	2.32	2.46	2.50	2.43	2.52	2.40	2.46	2.50	2.49	2.53	2.42	2.41	2.50	2.73	**3.11**
Dillapiol	12.28	12.22	12.23	12.31	13.60	14.26	14.45	15.30	15.43	15.49	15.80	15.78	15.95	16.56	**16.78**
Alpha-Phellandrene	1.38	1.33	1.39	1.36	1.37	1.41	1.33	1.31	1.20	1.43	1.51	1.52	1.44	1.42	1.52
*p*-cymene	–	–	–	–	–	–	–	–	–	–	0.10	0.20	0.22	0.18	0.15
Dihydrocarvone, trans-	0.23	0.29	0.21	0.30	0.22	0.20	0.29	0.29	0.31	0.19	0.19	0.19	0.19	0.26	0.19
6-Octadecenoic acid, methyl ester, (Z)-	1.56	1.71	1.16	1.13	1.33	1.66	1.71	1.17	1.09	1.63	1.36	1.31	1.41	1.40	1.64
Anethole	0.59	0.42	0.29	0.31	0.32	0.46	0.42	0.38	0.40	0.37	0.44	0.32	0.38	0.30	0.42
n-Hexadecanoic acid	1.61	1.10	1.32	1.46	1.06	1.31	1.24	1.24	1.44	1.74	1.01	0.10	0.15	0.21	0.19
Beta- Phellandrene	0.41	0.35	0.42	0.39	0.47	0.31	0.24	0.32	0.44	0.43	0.21	0.20	0.19	0.18	0.20
Sabinene	0.30	0.29	0.32	0.31	0.32	–	–	–	–	–	–	–	–	–	–
Alpha,2-dimethylstyrene	–	–	–	–	–	–	–	–	–	–	0.09	0.10	0.11	0.10	0.12
Cuminyl alcohole	–	–	–	–	–	–	–	–	–	–	0.10	0.11	0.20	0.23	0.26
Linalool	–	–	–	–	–	–	–	–	–	–	tr	tr	0.07	0.08	0.09
Total	91.97	92.24	91.98	92.18	94.79	93.47	96.18	97.97	99.34	98.91	94.23	95.59	96.60	97.76	99.39

Significant values are in bold.

– Not detected, tr: trace (< 0.01%).

Moreover, the results of this research indicated the chemical composition of dill seed essential oil is influenced by biochar-related treatments under non-saline and saline conditions. Application of biochar treatments, especially BNCs increased the major chemical compositions of the dill seed essential oil. The highest limonen content (27.32%) was obtained from the plants treated with biochar-based nanocomposites of zinc under moderate salt stress. The highest carvone (53.77%), dillapiole (16.78%), and apiol (3.11%) were recorded for combined use of biochar-based nanocomposites (BNC-FeO + BNC-ZnO) under high level of salinity. Moreover, the linalool, cuminyl alcohol, *p*-cymene, and alpha,2-dimethylstyrene were increased by application of biochar-related treatments under high salinity.

### Antioxidant activity (IC_50_)

The antioxidant activity of the seed essential oil was significant for the interaction of salt stress and nanocomposites (Fig. 2b). The IC_50_ values reveals the antioxidant activity of essential oil; the lower IC_50_ value, the higher antioxidant activity. The essential oils of differentially treated plants showed a considerable DPPH-inhibitory effect (antioxidant activity), with the IC_50_ values ranging from 14 up to 32 µg ml^−1^. The antioxidant capacity of seed essence was increased with rising salinity levels. As lower IC_50_ value indicates higher antioxidant activity, the highest antioxidant capacity (21 µg ml^−1^) was achieved at high level of salinity (12 dS m^−1^). The high level of salinity in comparison with moderate salinity and non-saline conditions increased the antioxidant activities by about 32% and 34%, respectively. Biochar and particularly nanocomposites showed a better effect on antioxidant activity under high level of salinity, compared with moderate salinity and non-saline conditions. Under non-saline condition, the biochar-based nanocomposites increased antioxidant activity of the essential oil. At moderate salinity (6 dS m^−1^), the highest values of antioxidant activity were recorded for plants under BNC-FeO and BNC-FeO + BNC-ZnO treatments, compared to pristine biochar. The combined form of nanocomposites was the superior treatment for enhancing antioxidant capacity (reducing the IC_50_ value) of plants under high level of salinity. The combined use of BNCs enhanced the antioxidant activity by about 34% under high level of salinity (12 dS m^−1^).

## Discussion

Salinity has different negative impacts on plants that cause a decrement in growth and production. Salt ions toxicity in the plant tissues can upset osmotic regulation and nutrient balance^6^. The high surface area, CEC, and porosity of BNCs under composition processes are important factors in controlling sodium uptake by plants that was reflected in high sodium sorption capacity values in biochar-based nanocomposites (Table 1). The reduction of sodium uptake and enhancement of nutrient availability by BNCs^6^, enhanced the iron and zinc contents of plant cells under salt stress (Fig. 1), overcoming the deficiencies of these nutrients. Increasing the antioxidant activity of essential oil by BNCs (Fig. 2b) can also play a role in mitigating salt toxicity. Our previous results revealed the beneficial effects of biochar-based nanocomposites on improving physiological performance of dill plants under salt stress through reducing Na uptake, and enhancing nutrient bioavailability, soil cation exchange capacity, leaf water content, and photosynthetic pigments^8,29^.

The quantity and quality of essential oils in medicinal plants can be influenced by environmental stresses and nutrient availability. Despite a reduction in plant productivity, different results have been reported on the variation of secondary metabolites of medicinal plants in response to salinity levels^28,30,31^. Increasing essential oil content at high level of salinity (Fig. 2a) was supported by the previous reports on *Mentha spicata*^32^, *Coriandrum sativum*^33^, *Lallemantia iberica*^34^, and *Ocimum basilicum*^30^. The increment of essential oil can be related to a higher density of oil glands as a consequence of the reduction in leaf area under salinity^1,35^. Salt toxicity can indirectly affect essential oil production through changes in assimilate partitioning during the plant growth and differentiation processes^31^. Additionally, a decline of the primary metabolites under saline conditions provides the intermediary products for an improvement in secondary metabolites synthesis^35^. Although biochar-related treatments had no significant effect on increasing the essential oil percentage, the effects of these treatments with particular physicochemical properties (Table 1), minimizing the Na uptake and maximizing the levels of available nutrients (Fig. 1), can improve the seed and essential oil yield of this plant under salinity^8,29^.

Similar to the previous studies^24,25^, limonene, carvone, and dillapiol in particular were identified as the main components of the essential oil extracted from dill seeds. Salt stress can alter the content of different essential oil components by changing biosynthetic processes^31^. In fact, enhancing in these secondary metabolites are an important part of plant defense system against environmental stresses. Increasing monoterpenes formation such as carvone and dillapiole due to salinity (Table 2) is dependent on terpene synthesis that is related to developmental and stress-related processes^36^. Under salt stress, disruptions in the ionic balance and enzymatic function affect many metabolic pathways and terpenoids metabolism^37,38^. At high level of salinity, dill seed essential oil was distinguished from other stress levels by the presence of alpha,2-dimethylstyrene, cuminyl alcohol, *p*-cymene, and linalool compounds (Table 2). The variation in essential oil profile can be attributed to the salt concentration and tolerance ranges of plants^30,38^. Indeed, variations of essential oil components are affected by changes in physiological functioning of plants and the induction of the specific enzymes involved in the biosynthesis of these compounds by salinity^37–39^. In response to salt stress, plants adapt through a number of mechanisms, such as salicylic acid (SA) signaling^28,29,35^. Salicylic acid is an efficient stimulant for secondary metabolite production that involves in the synthesis of terpenoids^40^. Enhancing salinity levels caused an increase in the content of important constituents including carvone, apiol, dillapioll, and alpha-phellandrene (Table 2) that can be attributed to increment of endogenous SA level. Declining limonene and enhancing carvone and *p*-cymene by salinity (Table 2) was the result of changing the chemotype of dill plants from limonene, as a precursor of carvone and *p*-cymene^41^. As a result, carvone is produced from limonene. In this process, GPP (geranyl pyrophosphate, precursor of the monoterpenes) is converted to limonene by a monoterpene synthase. Then, limonene is either stored in the essential oil ducts or oxidized to trans-carveol by a cytochrome P450-dependent hydroxylase. Then, trans-carveol is oxidized to carvone by a NAD^+^ or NADP^+^ utilizing dehydrogenase and stored in the essential oil ducts^42^.

The cytochrome P450 enzymes contain an active heme iron center, play a crucial role in the biosynthesis of secondary metabolites, antioxidants, and phytohormones in higher plants^43,44^. Therefore, application of iron-enriched biochar (BNC-FeO) can positively affect these chemicals by providing iron ions. In addition, geranyl pyrophosphate is a precursor to monoterpenes as the major constituents of dill essential oil. The GPP is produced by combination of DMAPP (dimethylallyl pyrophosphate) and IPP (Isopentenyl pyrophosphate), which is catalyzed by geranyl diphosphate synthase through methylerythritol phosphate pathway^45^, that could be improved by increasing P availability in biochar-related treatments (Table 1).

Biochar-based nanocomposites not only provide the essential nutrients for plants, but also act as carriers of nutrients that postpone their availability for plant uptake^6,16^. Enriched biochars with micronutrients such as Zn and Fe can help to better nutrient availability for specific enzymes involved in the synthesis pathways of constituents^8,31^. Iron and zinc as cofactors for Fe/Zn requiring enzymes are needed at various biosynthetic pathways^46,47^. Zinc acts as a structural or regulatory cofactor associated with saccharides metabolism which are a source of energy for terpenoid synthesis^48^. Moreover, CO_2_ and glucose are sources of carbon for monoterpenes biosynthesis. Considering the role of zinc and iron in photosynthesis^49^, biochar-based nanocomposites of iron and zinc can improve biosynthesis and concentration of secondary metabolites.

In summary, the addition of nanocomposites, especially in combined form to the soil is the most suitable method for obtaining higher ratios of carvone, apiol, and dillapiol, whereas BNC-Zn is preferable for obtaining particular components such as limonene (Table 2). The terpenoids such as limonene and carvone are produced from geranyl diphosphate, which is an intermediate in the mevalonate pathway. These monoterpene's synthesis pathway and their substrates require different nutrient supplies^50^. Moreover, nutrients can induce the expression of relevant genes of the monoterpene synthases, thereby altering individual compounds of essential oil. These compounds (limonene, carvone, and dillapiol) as the most detected in biochar-related treatments, especially in nanocomposites of iron and zinc (Table 2) are widely used in various industries as antifungal, antibacterial, insecticidal, antioxidant, and anti-inflammatory^41^.

Variation in main constituents of essential oil under salinity can lead to changes in antioxidant properties (Fig. 2b). Terpenoids are the most abundant phenolic compounds of essential oil (Table 2), because of their redox potential such as free radical scavenging activity, singlet oxygen quenching capacity, and hydrogen donors' activity are associated with the antioxidant activities^35,51,52^. It was reported that some monoterpenes including carvone and limonene contribute to the antioxidant activity of essential oil. Carvone as a phenolic compound and the main constituent found in dill oil (Table 2) has great antioxidant potential^51^. The increment of these compounds leads to a high antioxidant capacity of essential oil (Fig. 2b), which are also in connection with plant nutritional conditions (Fig. 1)^31^. These monoterpenes were impressively increased under biochar-related treatments. The highest scavenging of free radicals was achieved by combined form of BNC-FeO and BNC-ZnO, followed by BNC-ZnO under high level of salinity (Fig. 2b). This increment could be related to the composition of these products and their promoting effect on the nutrition and secondary metabolic pathways of the plants.

## Conclusion

Application of biochar-based nutritional nanocomposites of iron and zinc introduced as a more efficient form of biochar for lowering sodium uptake by the plants and improving nutrient bioavailability under salinity. The results showed that salinity and biochar-related treatments with the superiority of nanocomposites increased main monoterpenes and antioxidant activity, but essential oil content was only increased under high saline condition. Increasing main terpenoid compounds of essential oil as a result of biochar-related treatments can reflect the change in stress intensity and better nutritional status of plants. As a result, high level of salinity induced the production of some constituents such as alpha,2-dimethylstyrene, cuminyl alcohol, *p*-cymene, and linalool. Maximum limonene, carvone, apiol, and dillapioll were obtained by application BNC-ZnO and BNC-FeO + BNC-ZnO under salt stress. Furthermore, maximum antioxidant capacity of essential oil was recorded for combined form of nanocomposites (BNC-FeO + BNC-ZnO) under high level of salinity. Increasing the main constituents of the essential oil by BNCs could be considered a valuable aspect of biochar-based nanocomposites. Therefore, biochar-related treatments not only could mitigate the adverse effects of salinity on dill plants, but also can improve the secondary metabolic pathways leading to better essence quality.

## Materials and methods

### Providing biochar and biochar-based nanocomposites

Biochar was produced by pyrolysis of woody pruning residues of apple tree branches (as the major agricultural residue in the region). Pyrolysis was performed using a muffle furnace at 350 °C (at a rate of 5 °C/min) for 2 h under hypoxic condition^53,54^. After crushing into a powder, the resulted biochar was sieved using a 2 mm mesh screen and used for the production of biochar-based nanocomposites. The biochar was mixed with FeCl_3_·6H_2_O (10 mM) and ZnCl_2_ (10 mM) at a ratio of 1 g:10 ml^−1^ and stirred for 4 h. The mixed materials were oven-dried at 80 °C for 5 h and then heat-treated at 350 °C (at a rate of 5 °C/min)^55,56^ The physicochemical properties of biochar and biochar-based nanocomposites (BNCs) are presented in Table 1.

### Characteristics of biochar-related treatments

The elemental composition (C, H, N, and O) of biochar-related treatments was determined using a CHNOS elemental analyzer (Elementar-group, Hanau, Germany). Nutrients content of the biochar and BNCs were determined by extraction with BaCl_2_ and using ICP optical emission spectrometry (5800 ICP-OES Instrument, Hitachi, Tokyo, Japan)^20^. The amount of exchangeable ammonium was used to determine the cation exchange capacity (CEC)^57^. The pH values of biochar and BNCs were measured by a pH meter (Model: HI 99121, Hanna Instrument, USA) in a 1:2.5 (w/v ratio) biochar-water suspension. Brunauer–Emmett–Teller (BET) analysis was applied to determine specific surface area, pore size, and pore-volume distributions of materials using a Quantachrome Autosorb Automated Gas Sorption System^58^. The sodium sorption capacity of the biochar-related treatments was determined by a flame photometer (PEP7, Jenway, Dunmow, UK)^59^. The average particle sizes of Fe and Zn oxides within the biochar matrix were estimated by the Scherrer equation^60^. The thermal gravimetric analysis and differential scanning calorimetry (TGA/DSC) techniques^61^ were applied to estimate the thermal stability of biochar and biochar-based nanocomposites.

### Ethical approval

This experimental research upon plants complies with relevant institutional, national, and international guidelines and legislation.

## Experimental design

### Plant material and salt stress treatments

A factorial pot experiment based on a randomized complete block design with three replications was conducted in 2020 to assess the quantity and quality of essential oil in salt stressed dill plants. The pots were placed in a greenhouse with a day and night mean temperatures of 25 and 19 °C, air humidity of 39%, and a photoperiod of about 13 h.

Five biochar-related treatments [non-biochar, 30 g kg^−1^ biochar, 30 g kg^−1^ biochar-based nanocomposite of iron (BNC-FeO), 30 g kg^−1^ biochar-based nanocomposite of zinc (BNC-ZnO), and 15 g kg^−1^ BNC-FeO + 15 g kg^−1^ BNC-ZnO] were applied. The BNC-FeO and BNC-ZnO were separately produced and then a combined form of these nanocomposites (15 g kg^−1^ BNC-FeO + 15 g kg^−1^ BNC-ZnO) were prepared. The experimental soil (Table 1) of each pot (25 × 25 cm, capacity of 6 kg soil) was well mixed with biochar and BNCs according to the treatments.

Three levels of salinities [1.6 dS m^−1^, 6, and 12 dS m^−1^ as non-saline and moderate and high saline conditions, respectively] were adjusted by sodium chloride, sodium sulfate, calcium chloride, and magnesium sulfate with a molar ratio of 4:2:2:1. Different levels of salinity were selected based on a previous report on salt stress tolerance of dill plants^23^. The seeds of dill (Tabriz ecotype) were sown in 45 pots and 5 more pots with biochar treatments were kept unsown for checking water status of the similar sown pots. After sowing dill seeds, these solutions were added to the pots according to salinity treatments to achieve 100% field capacity. The weights of unsown pots before and after addition of saline solutions were recorded. The water losses from the pots were regularly compensated by tap water in all pots. For plant nutrition, 2 g of a fertilizer (Master 20-20-20-Valagro-Italy) was dissolved in 1 L of tap water (with an EC of 0.6 dS m^−1^ and a pH of 7.1) and added to the pots. After seedling establishment, the plants were reduced to seven plants per pot.

At full flowering stage (75 days after the sowing), two plants from each pot were removed and washed with distilled water for measuring cations (after oven-drying at 70 °C for 48 h). At maturity, remaining plants from each pot were harvested and the seeds were detached from the umbels to determine essential oil content and composition and antioxidant activity.

### Determination of sodium and nutrient contents

The dill plant tissues were dry-ashed for 7 h at 550 °C and then the ashes were digested in 5 ml HNO_3_. The samples were diluted in 45 ml distilled water within a volumetric flask and analyzed for sodium and mineral nutrients content (mg g^−1^ dry weight) with atomic absorption spectrophotometer (Shimadzu model: AA 7000, Kyoto, Japan)^62^.

### Essential oil extraction

The powdered of seeds were hydro-distilled for 3.5 h using a Clevenger apparatus according to Rostaei et al.^47^. The essential oils were dehydrated with anhydrous sodium sulphate and stored in a refrigerator at 4 °C.

### Essential oil composition

A GC–MS (Agilent 6890 N) system with a HP-5MS agilent column (30 m × 0.25 mm i.d., 0.25 μ film thickness) was used to quantify essential oil constituents of dill seeds. The injector temperature was 280 °C. The oven temperature was held at 50 °C for 5 min, then it was heated up to 280 °C at 3 °C/min, and remained constant for 7 min. Helium gas at a flow rate of 1.0 ml min^−1^ was used as carrier. The chemical components of the essential oil were determined by comparison of their mass spectra with those reported in the Wiley 5 library or with mass spectra from literature^47^.

### Antioxidant activity

The 2,2-diphenyl-1-picrylhydrazyl (DPPH) was used for free radical-scavenging activity test^63^. 20 µl of extracted essential oil were added to 100 µl of 0.5 mM DPPH solution in methanol and the mixture was diluted to achieve a final volume of 200 µl. The mixtures were kept at room temperature (25 °C) for 15 min and then the absorbance was recorded at 517 nm using a spectrophotometer. The DPPH inhibition of the samples was calculated as:

$${\text{DPPH}}\,\,{\text{inhibition}}\,\,\left( \% \right)\,\, = \,\,\left\{ {\left( {{\text{A}}_{0} - {\text{A}}_{{1}} } \right)/{\text{A}}_{0} } \right\}\, \times \,{1}00$$

where A_0_ and A_1_ are the absorbance of control and the DPPH radical in the presence of the plant essential oil, respectively. The sample concentration providing 50% inhibition (IC50) was determined by plotting the graph of DPPH inhibition percentage against sample concentration.

### Statistical analysis

Analysis of variance (Two-Way ANOVA-factorial arrangement on the basis of randomized complete block design with three replicates) was performed using SAS software (version 9; SAS Institute; USA). The means of the data were compared by the Duncan’s Multiple Range test at the 5% probability level.

## Data Availability

The necessary information is available from the corresponding author on reasonable request.
